# Case report of IgG4-related appendiceal disease: A challenging disease

**DOI:** 10.1097/MD.0000000000020588

**Published:** 2020-06-19

**Authors:** Adriano Basso Dias, Natally Horvat, Maria Dirlei Begnami, Emerson Shigueaki Abe, Publio Cesar Cavalcante Viana, Marcel Cerqueira Cesar Machado

**Affiliations:** a Department of Radiology; b Department of Pathology; c Department of Surgery, Hospital Sírio-Libanês, São Paulo, Brazil.

**Keywords:** appendicitis, appendix, case report, immunoglobulin G4-related disease, multidetector computed tomography

## Abstract

**Rationale:**

Immunoglobulin G4 (IgG4)-related disease is an increasingly recognized immune-mediated entity that can affect virtually every organ system. Depending on the location of the disease, it can present a wide range of clinical manifestations and even mimic malignancies. Appendiceal involvement in patients with IgG4-related disease is particularly rare and very few cases are reported in the literature.

**Patient concerns:**

We report a case of IgG4-related appendiceal disease in a 42-year-old woman who presents with a subacute onset of right lower quadrant abdominal pain.

**Diagnosis:**

Abdominal computed tomography showed a markedly enlarged appendix, raising the concern of malignancy. The diagnosis of IgG4 appendiceal disease was confirmed by postoperative histopathologic and immunohistochemical examination.

**Interventions:**

The patient underwent right hemicolectomy.

**Outcomes:**

After the surgery, the patient had an uneventful recovery and reported a resolution of her symptoms. The serum IgG4 was revaluated 5 days after surgery and returned to its normal values. At the 3-year follow up, the patient had no recurrence of symptoms and her imaging exams remain unremarkable.

**Lessons:**

This study reports the fifth case of IgG4-related appendiceal disease. Increasing awareness of this condition may influence the management of these patients, once patients with IgG4-related disease should be monitored after treatment, due to the risk of recurrence or involvement of other organs.

## 1 Introduction

Immunoglobulin G4 (IgG4)-related disease is an increasingly recognized immune-mediated entity that includes a group of conditions with specific clinical, serologic, and pathologic features, which is characterized by a significant steroid responsiveness.^[1–3]^ The disease was initially described in the pancreas, but since it has been described in virtually every organ system, including the biliary tree, salivary glands, periorbital tissues, kidneys, lungs, lymph nodes, aorta, breast, prostate, thyroid, and skin.^[1,2]^ Most of the lesions present as organ enlargement or nodular lesions mimicking infectious or malignant lesions. Histologically, these lesions consist of infiltration of lymphocytes and IgG4-positive plasma cells and fibrosis.^[4]^ IgG4-related appendiceal disease is extremely unusual. To the best of our knowledge, there are only 4 cases that describe appendiceal disease associated with increased IgG4 plasma cells^[5–8]^ and there is only one that fulfills all pathological diagnostic criteria.^[8]^

The aim of this study is to describe the fifth case of IgG4-related appendiceal disease, but the second one that fully satisfied all the pathological criteria, and review the literature. The patient has provided informed consent for publication of the case.

## 2 Case report

A previously healthy 42-year-old woman was admitted in the emergency department with a 1-week history of right lower quadrant abdominal pain. Physical examination revealed a painful mass in the right lower quadrant with no rebound or guarding, normal bowel sounds, and no hepatosplenomegaly. Physical examination was otherwise normal, and the initial laboratory tests were normal.

**Figure 1:**
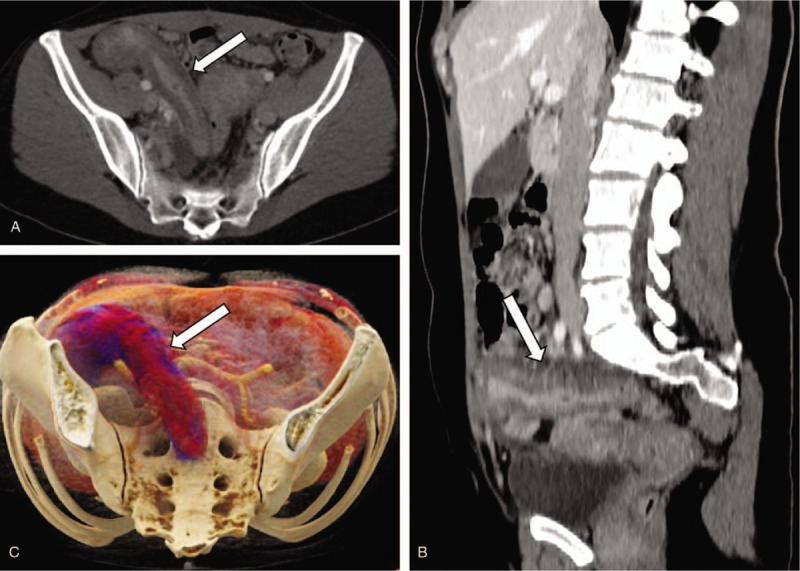
A 42-year-old woman, with IgG4-related appendiceal disease. Contrast-enhanced CT in axial plane (A), oblique sagittal plane (B), and 3D cinematic volume rendering reconstruction in axial plane (C) demonstrate the appendix with marked dilation, thickened walls, and mucosal hyperenhancement (arrows). CT = computed tomography, IgG4 = immunoglobulin G4.

Abdominal computed tomography (CT) scan with intravenous iodinated contrast media was requested to evaluate his symptoms. CT scan showed a marked enlargement, thickening, and mucosal hyperenhancement of the appendix (Fig. 1). There was no significant appendiceal distention, appendicolith, periappendicular fat stranding, or free intraperitoneal fluid. No additional abnormalities were detected on CT, including lymph node enlargement.

**Figure 2:**
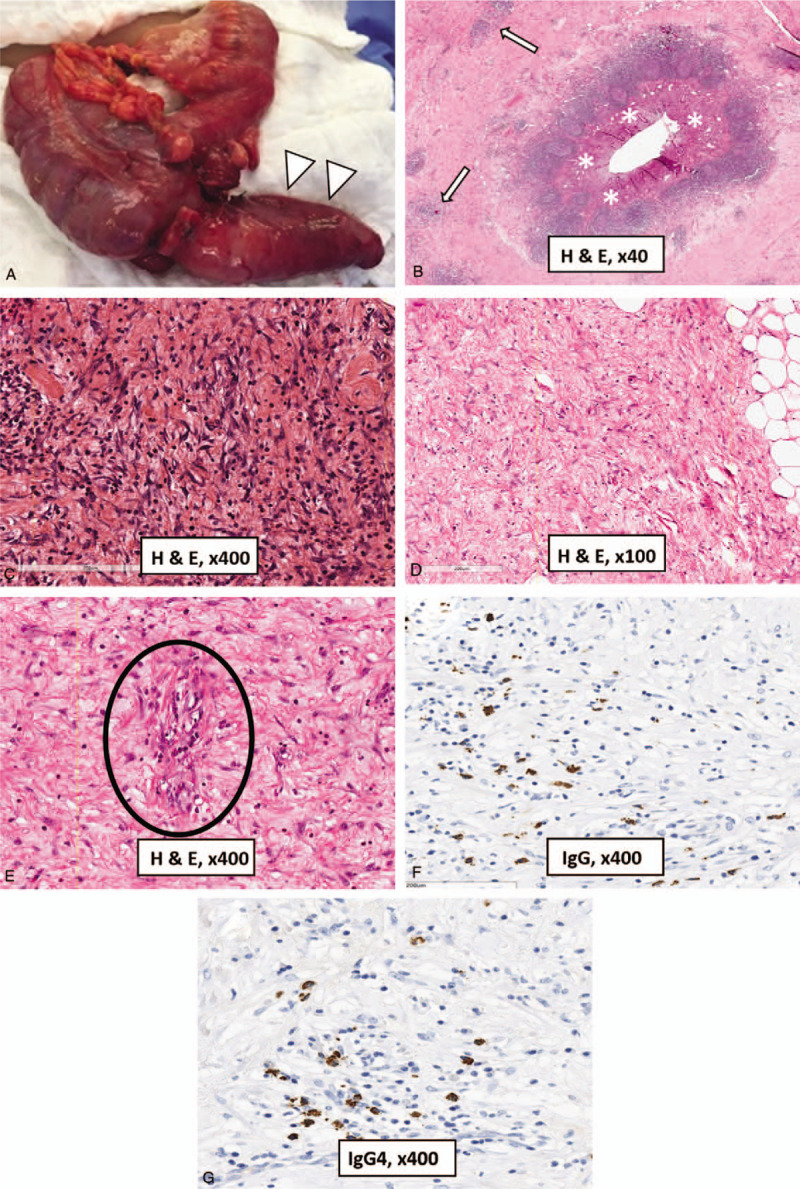
Surgical specimen and pathologic findings after right hemicolectomy. Surgical specimen (A) shows marked enlargement of the appendix (arrowheads). Histological evaluation (hematoxylin and eosin staining) demonstrates the appendiceal mucosa (asterisks) relatively unremarkable (B). There is a transmural and chronic fibroinflammatory process composed of dense lymphoplasmacytic infiltrate (arrows) and spindle cell proliferation in the appendiceal wall (B). There are areas showing storiform fibrosis associated with dense lymphoplasmacytic infiltration in the subserosa of appendix (C) and in the mesoappendix (D). Vein (inside the circle) completely obliterated by aggregated inflammatory cell infiltration (obliterative phlebitis) is also noted in the appendiceal wall (E). Immunohistochemical analysis shows IgG (F) and IgG4+ plasma cells (G) demonstrating increased numbers of IgG4+ plasma cells (up to 21/high power field, with the IgG4/IgG ratio >40%).

The patient underwent an open right hemicolectomy (Fig. 2A). A frozen section was performed with the diagnosis of a benign lesion. Gross examination showed enlargement of the cecum and the appendix with irregular wall thickening and submucosal sclerosis. The surgical specimen was fixed in 10% buffered formalin, and then it was processed routinely. The sections were stained with hematoxylin and eosin. On histopathological evaluation, the appendiceal wall was thickened and the mucosa was unremarkable with no evidence of acute appendicitis (Fig. 2B). There was a patchy dense transmural lymphoplasmacytic inflammatory process involving the submucosa, the muscular layer, the subserosa, and the periappendiceal tissue intermixed with spindle cell proliferation. Additionally, areas of dense storiform fibrosis and sclerosis were identified in association with obliterative phlebitis (Fig. 2C–E). The remaining right colon appeared histologically unremarkable.

Immunostaining was performed according to standard protocols using avidin–biotin complex labeled with peroxidase. Appropriate positive and negative controls were run concurrently for all the markers tested. The inflammatory cells were composed of mixed reactive population of T and B cells with plasmacytosis. There were mostly CD3-positive lymphocytes, whereas CD8 and CD20-positive lymphocytes were less numerous. Immunostaining for IgG and IgG4 (Fig. 2F and G) demonstrated an increased numbers of IgG4-positive plasma cells (up to 21 plasma cells/high power field) and the ratio IgG4/IgG>40%. Based on clinical, radiological, and pathological findings, a diagnosis of IgG4-related appendiceal disease was made. After this histological diagnosis, the serum IgG4 concentration was analyzed using the blood sample collected before the surgery. There was a slight elevation of serum IgG4 concentration (180 mg/dL; normal value < 135 mg/dL).

After the surgery, the patient had an uneventful recovery and reported a resolution of her symptoms. The serum IgG4 was revaluated 5 days after surgery and returned to its normal values. At the 3-year follow-up, the patient had no recurrence of symptoms and her imaging exams remain unremarkable.

## 3 Discussion

IgG4-related disease (IgG4-RD) is a newly recognized immune-mediated disorder that can affect various organs and usually presents as tumefactive lesions that mimics malignant conditions.^[2,4]^ However, reports on appendiceal involvement are very limited. To the best of our knowledge, up to now there are only 4 cases that describe appendiceal disease associated with increased IgG4 plasma cells.^[5–8]^ The pathophysiology of IgG4-RD remains poorly understood; however, type 2 T-helper cell, which regulates T cell cytokines, and B cell activating factor, have been suggested to be associated with the development of the disease.^[9]^

Gastrointestinal IgG4-related diseases are difficult to be diagnosed by clinical and radiological findings alone, owing to its nonspecific presentation. Most of reported cases were diagnosed only after surgical resection.^[10–14]^ Pancreas was the first and is the most frequent digestive organ to be involved in IgG4-RD followed by bile ducts.^[15]^ IgG4-related lesions of the small bowel and colon are extremely rare. Only 2 cases of solitary IgG4-RD at the cecum and sigmoid colon have been reported, and only 1 case at the ileocecal region mimicking malignancy has been reported.^[16]^ The involvement of the appendix is also rare.^[5–8]^ Patients with IgG4-RD are at risk to have recurrent disease or involvement of other organs over time; furthermore, lymphomas can occur.^[2,4,8]^ Considering that these patients should be followed up at regular visits.

According to the Consensus created by the International Committee on IgG4-related disease held in 2011, the 3 major histopathological features associated with this condition are dense lymphoplasmacytic infiltrate, storiform fibrosis, which is characterized by an irregularly cartwheel-like fibrotic pattern, and obliterative phlebitis, in combination with increased IgG4+ plasma cells infiltrating the tissue.^[2]^ The cutoff threshold appears to vary by organ due to the nature of fibrosis and limited published data. The International Committee has proposed organ-specific cutoff, which ranges from >10 per high—power field to >200 per high-power field.^[2]^ However, it is important to highlight that appendiceal involvement has not been included in the Consensus since there were no published case reports at that time. Therefore, specific cutoff value was not described for this organ. The Committee also considered IgG4/IgG plasma cell ratio a more powerful tool than the IgG4+ plasma cell counts in establishing the diagnosis of IgG4-related disease. An IgG4/IgG ratio >40% is established as a comprehensive cutoff value in any organ.^[2]^ Other minor features such as eosinophilia, phlebitis without obliteration, increased serum IgG4, and response to steroids are also described.^[2]^ Prominent neutrophilic infiltration, granuloma, and necrosis are features that would exclude the diagnosis of IgG4-RD and increase the suspicion for infections, sarcoidosis, or granulomatous diseases.^[2]^

Table 1 summarizes the studies that described IgG4-related appendiceal disease, including our case.^[5–8]^ The average age of patients was 43 years, ranging from 20 to 57 years, and the majority was woman (4/5, 80%). All patients presented with right lower quadrant abdominal pain, 4 of them (80%) with an acute or subacute onset and 1 (20%) with a chronic presentation (4–5 months). The last patient had also weight loss complaint.^[7]^ Regarding imaging findings, CT showed appendiceal mass-forming lesions in 2 cases,^[6,8]^ and appendiceal enlargement in one of the previous reported cases.^[8]^ One study did not describe if preoperative radiological study was performed.^[5]^ All studies that described the imaging features on CT demonstrated no extra-appendiceal disease. Finally, only 1 case demonstrated slight periappendiceal inflammatory changes^[6]^ and also only 1 patient showed distension of the appendiceal lumen.^[7]^

**Table 1:**
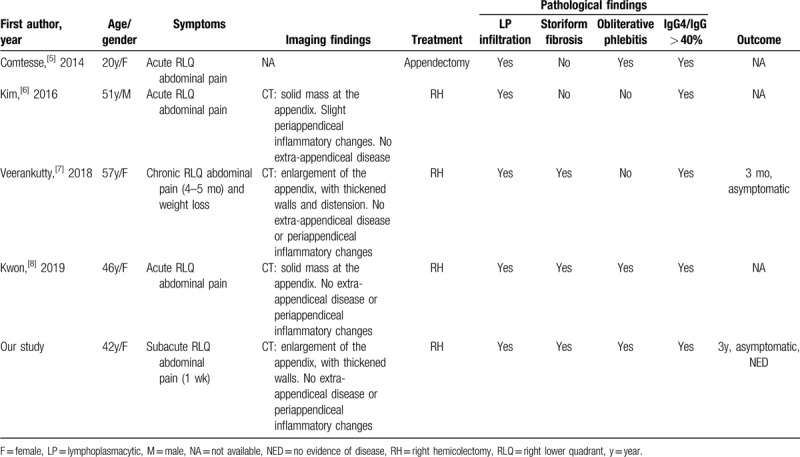
Summary of cases of IgG4-related appendiceal disease found in literature.

All patients underwent surgical treatment, most of them with right hemicolectomy (4/5, 80%), due to the possibility of malignancy, and one of them with appendectomy, since the presumptive diagnosis was acute appendicitis. The prognosis of this entity is not described in the literature due to its rarity. Apart from our study, only Veerankutty et al^[7]^ reported the patient follow-up, who was also free of disease and had good recovery from symptoms 3 months after surgery.

Besides our study, only Kwon et al^[8]^ reported a patient who satisfies the full spectrum of histological criteria recommended by the International Committee. We also report the first case with pre- and postoperative serum IgG4 level, demonstrating the reduction of its level after surgery. It‘s important to highlight that the pretreatment elevation of this serum immunoglobulin is not a sensitive criterion for the diagnosis of IgG4-RD, which can be normal in up to 40% of patients.^[2]^ Moreover, there is no evidence that the decrease of this serological marker in the postsurgical setting is a reliable criterion for the diagnosis of this disease.^[2]^ Therefore, the serum IgG4 level is not considered a major criterion for this condition (as those 3 histopathological features mentioned before, in combination with increased IgG4+ plasma cells in the surgical specimen).^[2]^ However, according to the Consensus, elevated serum IgG4 level (> 135 mg/dL) can be considered an additional criterion to suggest this diagnosis in patients with “Probable Histological Features of IgG4-Related Disease” (e.g., in cases with only a single histopathological feature).^[2]^ Our case fulfilled all 3 histopathological features as well as the immunohistochemical criteria (IgG4/IgG ratio > 40% in the tissue), thus the serum IgG4 concentration was not completely necessary in this case, but it helps to reinforce this diagnosis.

The main differential diagnosis of IgG4-related appendiceal disease on imaging is acute appendicitis and malignancies, including adenocarcinoma and neuroendocrine tumors. Although rare, IgG4-related appendiceal disease should be suspected when appendiceal involvement is disproportionately more intense than the periappendiceal inflammatory changes, which can be slight or even absent. Furthermore, when other findings of IgG4-RD are present, such as involvement of the pancreas, biliary tree, kidney, and/or retroperitoneum, the diagnosis appendiceal involvement should be considered. In these situations, a therapeutic test with steroids may be considered. However, the mass-like form of the disease is challenging to be diagnosed based solely on imaging features and a definitive diagnosis may require histopathology.

Recognition of this entity has an important clinical implication, considering that a steroid test can be performed before surgery in order to avoid an unnecessary surgical procedure. Increasing awareness of this condition may also influence the management of these patients, once patients with IgG4-related disease should be monitored after treatment, due to the risk of recurrence or involvement of other organs.

## Author contributions

Adriano Basso Dias: Manuscript writing.

Natally Horvat: Study concept and manuscript writing.

Maria Dirlei Begnami: Major role in the acquisition of data.

Emerson Shigueaki Abe: Critical revision of the manuscript for important intellectual content.

Publio Cesar Cavalcante Viana: Critical revision of the manuscript for important intellectual content.

Marcel Cerqueira Cesar Machado: Study concept, critical revision of the manuscript for important intellectual content.
